# Socio-Economic Resilience to Floods in Coastal Areas of Thailand

**DOI:** 10.3390/ijerph19127316

**Published:** 2022-06-14

**Authors:** Uma Langkulsen, Desire Tarwireyi Rwodzi, Pannee Cheewinsiriwat, Kanchana Nakhapakorn, Cherith Moses

**Affiliations:** 1 Faculty of Public Health, Thammasat University, Rangsit Campus, Pathum Thani 12120, Thailand; 2 United Nations Programme on HIV/AIDS Regional Support Team for Asia and the Pacific, Bangkok 10200, Thailand; rwodzidesire@gmail.com; 3 Geography and Geoinformation Research Unit, Department of Geography, Faculty of Arts, Chulalongkorn University, Bangkok 10330, Thailand; 4 Faculty of Environment and Resource Studies, Mahidol University, Nakhon Pathom 73170, Thailand; kanchana.nak@mahidol.edu; 5 Department of Geography and Geology, Edge Hill University, Ormskirk L39 4QP, UK; mosesc@edgehill.ac.uk

**Keywords:** socio-economic, resilience, floods, vulnerability, coping capacity

## Abstract

Krabi and Nakhon Si Thammarat are two coastal provinces in Thailand facing substantial threats from climate change induced hydrometeorological hazards, including enhanced coastal erosion and flooding. Human populations and livelihoods in these coastal provinces are at greater risk than those in inland provinces. However, little is known about the communities’ resilience and coping capacities regarding hydrometeorological hazards of varying magnitudes. The study conducted a quantitative socio-economic assessment of how people in Krabi and Nakhon Si Thammarat provinces manage and respond to hydrometeorological hazards, examining their resilience and coping capacities. This was a cross-sectional study based on secondary data collection on the social and economic dimensions of resilience, and a review of literature on coping mechanisms to hydrometeorological hazards within the study area. Measuring and mapping socio-economic resilience was based on the available data gathered from the social and economic dimensions, with existing or standard indicators on exposure and vulnerability applied uniformly across subdistricts. A combination of social and economic dimensions produced novel socio-economic resilience index scores by subdistrict, which were mapped accordingly for the two coastal provinces. The study also derived a coping capacity index scores by combining availability of skills or soft capacity and availability of structural resources or hard coping capacity. Socio-economic resilience index scores varied greatly amongst subdistricts. Combining the soft and hard coping capacities, the average score across districts in both provinces was 3 out of a possible 4, meaning that most of the districts were largely resilient. However, variations also existed by subdistrict. Few subdistricts in both Krabi and Nakhon Si Thammarat provinces had low coping capacity index scores between 1 and 2 out of 4. District averages of socio-economic resilience scores mask the variations at subdistrict level. More studies with rigorous methodologies at village or neighborhood level is needed to obtain a nuanced understanding of community resilience to hydrometeorological hazards.

## 1. Introduction

In recent years, hydrometeorological hazards have become more frequent and severe, with increasing loss of life and damage to livelihoods, property, and infrastructure [1]. Coastal areas in Thailand face recurring flooding situations resulting in communities and individuals adopting a range of coping mechanisms [2]. Coping capacity entails to the ability of people, organizations, and systems, using available skills and resources, to manage adverse conditions, risk, or disasters [3]. Following the floods in 2004, the Thai Red Cross, in partnership with other agencies, with funding from United Nations Development Programme, implemented a project in three provinces namely Nakhon Si Thammarat, Phatthalung, and Trang to strengthen the adaptive capacity of vulnerable coastal communities in Thailand to climate change related risks and weather events [4].

The action plans implemented in three provinces, including mud dredging along coastlines, community training, water management by community, alternative rice production techniques, community zoning, shoreline protection, etc. [4]. Individual coping strategies without collective action may not be effective solutions due to the occurrence of negative externalities if neighbors do not apply/maintain their own protection structures. Following the 2004 Tsunami, households in Ban Nam Khem community started collectively implementing regular tsunami drills, pick-up services for elderly people in the case of an emergency, or the planning of escape routes and tsunami shelters [5].

Recent studies have emphasized the need to understand community resilience to disasters [6]. Efforts to increase community resilience to natural hazards should be guided by the Sendai Framework for Disaster Risk Reduction 2015–2030 [7]. This starts with gaining a better understanding of disaster risk and strengthening disaster risk governance to manage the risk, instead of managing the disaster after it happens [8]. Exposure is defined as the situation of people, infrastructure, housing, production capacities, and other tangible human assets located in hazard-prone areas [9], whereas vulnerability is the conditions determined by physical, social, economic, and environmental factors or processes which increase the susceptibility of an individual, a community, assets, or systems to the impacts of hazards [10]. Resilience is defined as the ability of a system, community or society exposed to hazards to resist, absorb, accommodate, adapt to, transform, and recover from the effects of a hazard in a timely and efficient manner, including through the preservation and restoration of its essential basic structures and functions through risk management [11].

A previous study revealed that mapping urban resilience poses a challenge to reflect the systematic property of resilience [12]. Several studies showed disaster management approach in the mitigation, preparedness, response, and recovery phases using flood hazard map, hospital evacuation transportation model, livelihood vulnerability index (LVI), model suitability matrix, and investments in flood protection [13,14,15,16,17]. Different models have been used in the past to examine resilience to different hazards and disasters. One well-known approach is to improve comparative assessments of disaster resilience at the community level. The Disaster Resilience of Place (DROP) model was developed to address resilience to natural disasters [18]. The DROP model was used at the provincial level across Thailand. Results of the model suggest that while disaster resilience is generally higher in the more urbanized areas, communities located in rural areas in Thailand may not necessarily be less resilient to the impacts of disasters. The role of place in disaster resilience needs to be considered in any resilience model, as indicators need to capture the unique cultural and structural characteristics of the study area [6]. The assessment of resilience against socio-economic and health risks should be a concern in the urban resilience assessment [19].

There is a dearth of information with respect to two of the coastal provinces in Thailand namely Krabi and Nakhon Si Thammarat in terms of the resilience of the communities and their coping mechanisms to climate related hazards. The study sought to provide a quantitative socio-economic assessment of the two provinces to understand their vulnerability to hydrometeorological hazards of varying magnitudes; and to come up with socio-economic resilience index scores as well as some insights on the coping mechanisms being adopted. The main contributions this study makes relate to understanding the socio-economic resilience to floods, vulnerability, and coping capacity, as well as insights serve to guide future research on resilience to floods.

## 2. Materials and Methods

### 2.1. Study Area

**Figure 1:**
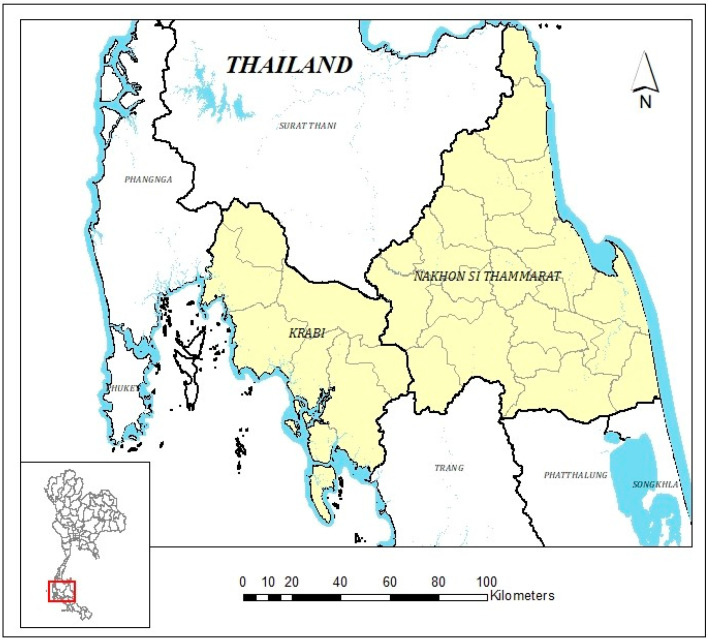
Map of study area.

A descriptive cross-sectional study was conducted focusing two coastal provinces in Thailand, namely, Krabi and Nakhon Si Thammarat, as shown in Figure 1. Krabi and Nakhon Si Thammarat provinces are in the southern parts of Thailand. Severe inundation during the rainy season and coastal erosion influenced the selection of the two provinces for data collection so that their coping mechanisms and resilience could be strengthened. The study area had a total of 245 administrative units, comprising 61 and 184 units in Krabi and Nakhon Si Thammarat provinces, respectively. In both provinces, subdistricts constitute the largest portion as shown in Table 1.

### 2.2. Data Collection

Secondary data reflecting the social and economic dimensions of resilience to hydrometeorological hazards were gathered from different departments and ministries. Data gathered focus on mapping the demographics of the study area, including exposure, vulnerability, and soft and hard coping capacities. The exposure sub-indicator is defined as the population density as shown in Table 2. The vulnerability identified the vulnerable groups among the affected population, including infants, children under the age of 5, elderly population, prisoners, orphans and homeless persons, disabled persons, people with underlying chronic diseases such as chronic kidney disease (CKD), chronic obstructive pulmonary disease (COPD), diabetes mellitus (DM), hypertension (HT), and stroke, and area of mangrove forest. Soft coping capacity identified the literacy rate, whereas hard coping capacity identified the hospital at subdistrict, district, and provincial levels, and telecommunication development.

### 2.3. Data Analysis

Simple descriptive analyses were conducted, focusing on the distribution of different variables within the two coastal provinces. The social and economic dimensions were then combined to produce socio-economic resilience index scores and maps for the two coastal provinces.

In this investigation, resilience to hydrometeorological hazards was considered a function of exposure, vulnerability, and coping capacity as shown in Table 2. Population density at subdistrict level was the only exposure variable and was measured on a scale from 0 to 5, with 5 representing the highest level of exposure. Several variables were considered under the dimension on vulnerability, and the same scale from 0 to 5 was applied, with 5 representing greatest level of vulnerability. Combining the exposure and vulnerability variables, we derived socio-economic resilience index scores from 0 to 45, with lower scores representing greater resilience.

Coping capacity referred to the availability of skills (soft coping capacities) and structural resources (hard coping capacities) to help communities manage and respond to hydrometeorological hazards. Based on readily available data, the only variable considered under soft coping capacity was literacy rate, and this was measured on a scale from 0 to 1, with 1 representing an area had a literacy rate greater or equal to 90%. A community’s capacity to cope requires continuing awareness amongst the population, hence, the inclusion of literacy rate. For the hard coping capacities, the study took into consideration the availability of a hospital at subdistrict, district/provincial levels as well as telecommunication development. Combining the soft and hard coping capacities, the study then derived and mapped the coping capacity index scores ranging from 0 to 4, with 4 representing the maximum possible coping capacity.

## 3. Results

### 3.1. Exposure and Vulnerability

**Figure 2:**
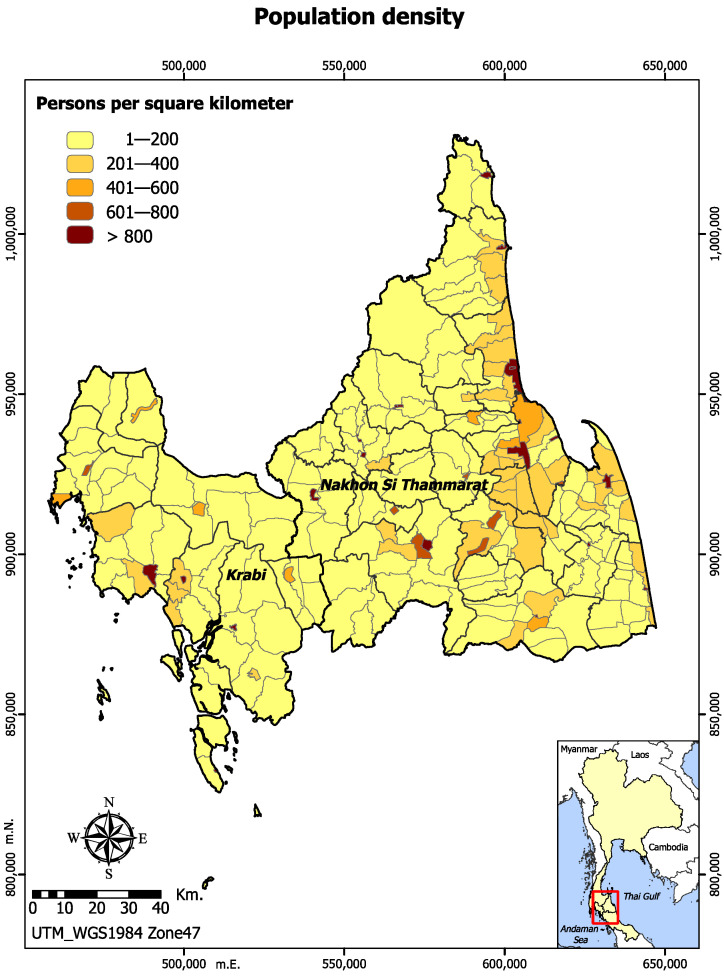
Population density in Krabi and Nakhon Si Thammarat, 2017.

As of 2017, most of the administrative units in both Krabi and Nakhon Si Thammarat had a population density of 1 to 200 persons per square kilometer as shown in Figure 2.

**Figure 3:**
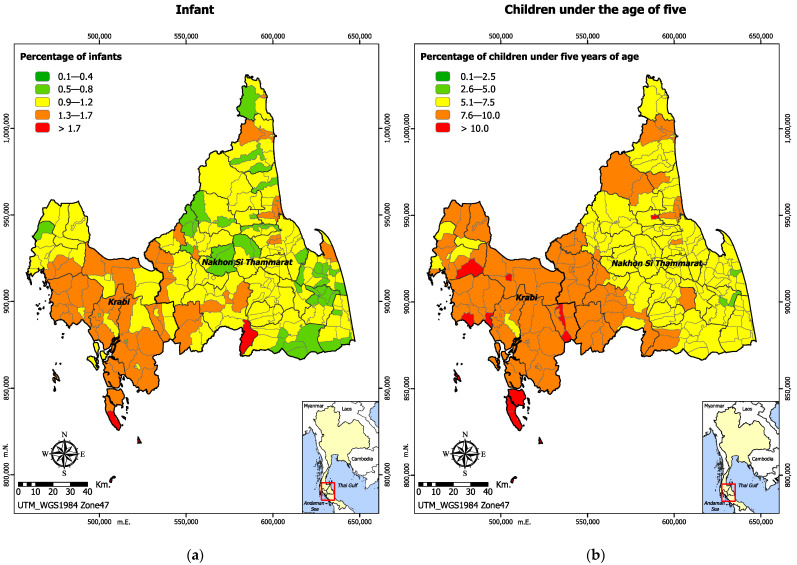

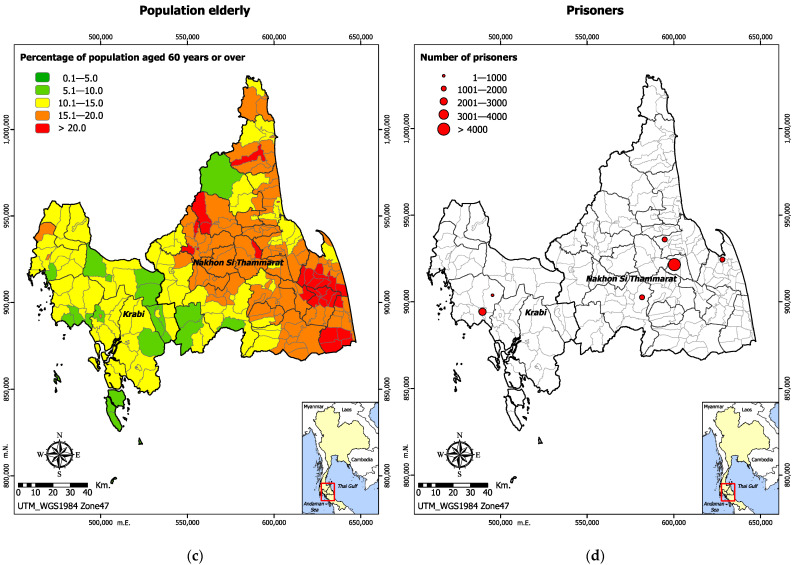

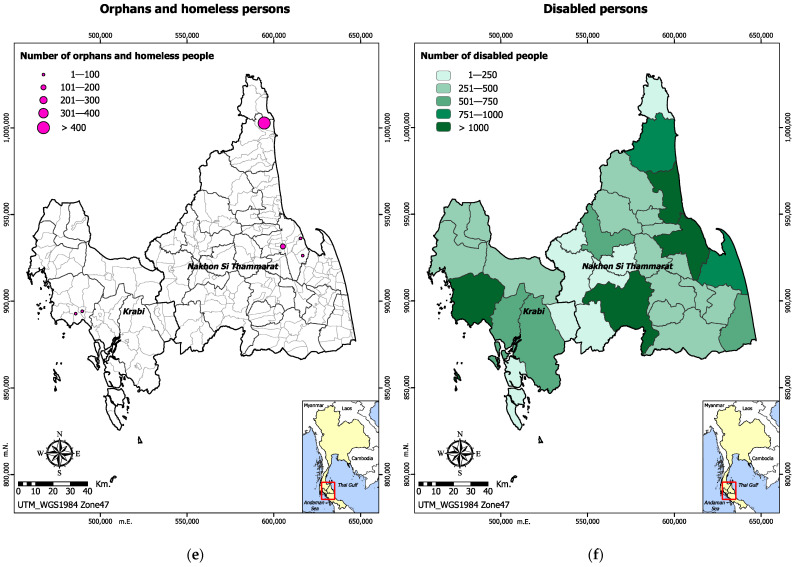

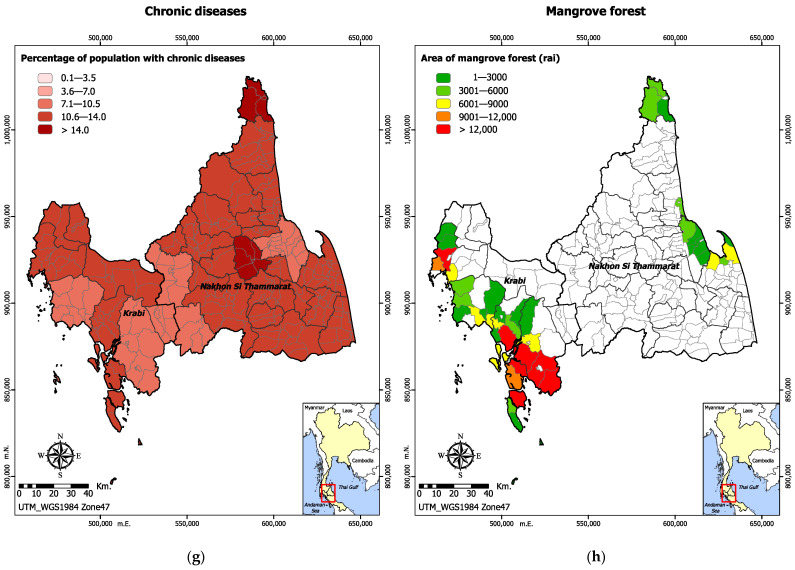
Vulnerability to natural hazards: (**a**) proportion of infants; (**b**) proportion of children under the age of five; (**c**) proportion of population aged 60 years and over; (**d**) number of prisoners; (**e**) number of orphans and homeless people; (**f**) number of disabled persons; (**g**) proportion of population with chronic diseases; (**h**) area of mangrove forest.

While the majority of subdistricts in Nakhon Si Thammarat had an infant population ranging from 0.9% to 1.2%, most subdistricts in Krabi had a higher proportion of infants, ranging from 1.3% to 1.7%, as shown in Figure 3a. Most subdistricts in Krabi had a higher proportion of children under five years of age compared to Nakhon Si Thammarat. For Nakhon Si Thammarat, subdistricts closer to the border with Krabi exhibited a similar pattern as shown in Figure 3b. Overall, most subdistricts in Nakhon Si Thammarat had a greater proportion of population aged 60 years and over, ranging from 15.1% to greater than 20% as shown in Figure 3c.

Not all subdistricts have prisons, but, overall, there were more prisoners in Nakhon Si Thammarat than in Krabi, as shown in Figure 3d. Numbers of orphans and homeless people were very small in both provinces. Only one subdistrict in Nakhon Si Thammarat had more than 400 orphans and homeless people, as shown in Figure 3e. All subdistricts in both provinces had disabled persons. Only one district (Mueang Krabi) in Krabi compared to three districts (Tha Sala, Mueang Nakhon Si Thammarat, and Thung Song) in Nakhon Si Thammarat had at least 750 persons living with disabilities as shown in Figure 3f. Chronic diseases were prevalent in both provinces. Most subdistricts in both Krabi and Nakhon Si Thammarat provinces had more than 7% of their population living with a particular chronic ailment as shown in Figure 3g. Between the two provinces, Krabi had the largest area and the greatest number of districts covered by mangrove forests as shown in Figure 3h.

### 3.2. Resilience to Disasters

Based on data collected, variables of exposure, vulnerability, soft coping capacity, and hard coping capacities were developed in a scale in each of 61 (48 subdistricts, 12 subdistrict municipalities, 1 town municipality) and 184 (131 subdistricts, 49 subdistrict municipalities, 3 town municipalities, 1 city municipality) administrative units in Krabi and Nakhon Si Thammarat provinces.

Socio-economic resilience index scores were derived by adding the exposure and vulnerability scores, with a higher score suggesting less resilience to hydrometeorological hazards. Out of a possible total resilience score of 45, Mueang Krabi and Nuea Khlong districts had the highest score of 23, indicating least resilience. Lam Thap district had the greatest resilience with a score of 14 out of 45. However, district averages masked the variations among subdistrict scores. The subdistrict with the least resilience in terms of exposure and vulnerability to water related disasters was Mueang Krabi town municipality and Khlong Khanan subdistrict.

In Nakhon Si Thammarat, district average socio-economic resilience index scores for exposure to water related hazards mask the variations among subdistrict scores. All districts had average scores less than half of the maximum possible 45. However, there were several subdistricts with scores above 20 out of 45. Sichon district was the least resilient, and Thung Sai Subdistrict Municipality had the highest score of 23. The most resilient district was Bang Khan, with a socio-economic resilience index score of 14 out of 40.

### 3.3. Coping Capacities

**Figure 4:**
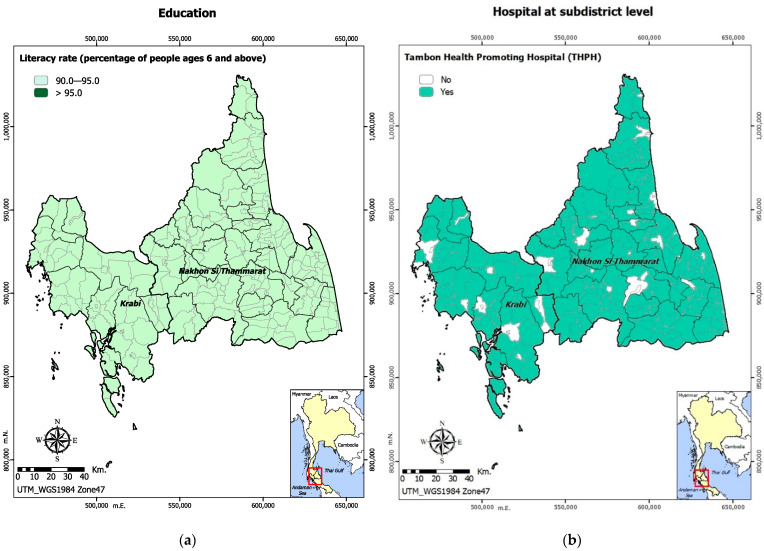

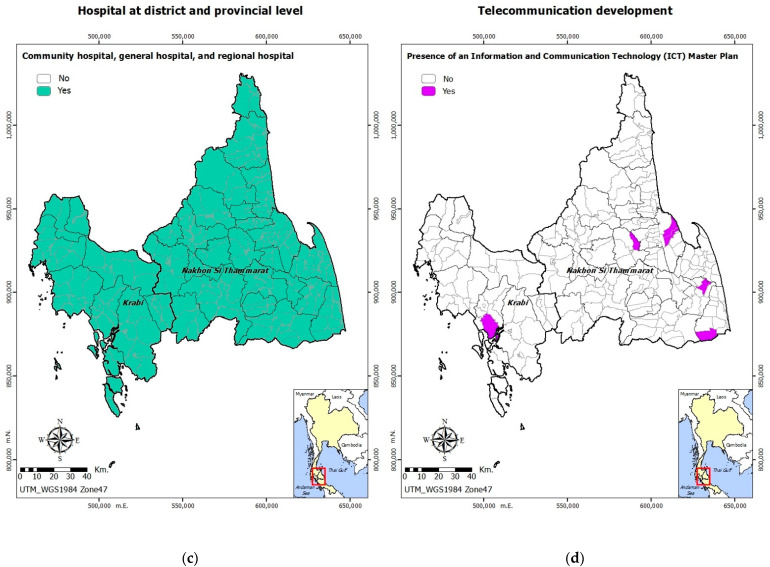
Soft and hard coping capacities: (**a**) literacy rate among people aged 6 years and above; (**b**) availability of hospitals at sub-district level; (**c**) availability of a hospital at district and provincial level; (**d**) presence of Information and Communication Technology (ICT) Master plans.

Literacy rate was included in this assessment as the only soft copying capacity. In both Krabi and Nakhon Si Thammarat provinces, all subdistricts had a literacy rate between 90% and 95% among people aged 6 years and above, as shown in Figure 4a.

In both provinces, Tambon Health Promoting Hospitals were available in most of the Tambon areas, as shown in Figure 4b. All subdistricts in both provinces were at least covered by either a functional hospital, either at community level, district level, or regional level, as shown in Figure 4c. Very few subdistricts in both provinces had Information and Communication Technology (ICT) master plans in place; however, Nakhon Si Thammarat had more Tambon areas with the master plans than Krabi, as shown in Figure 4d.

**Figure 5:**
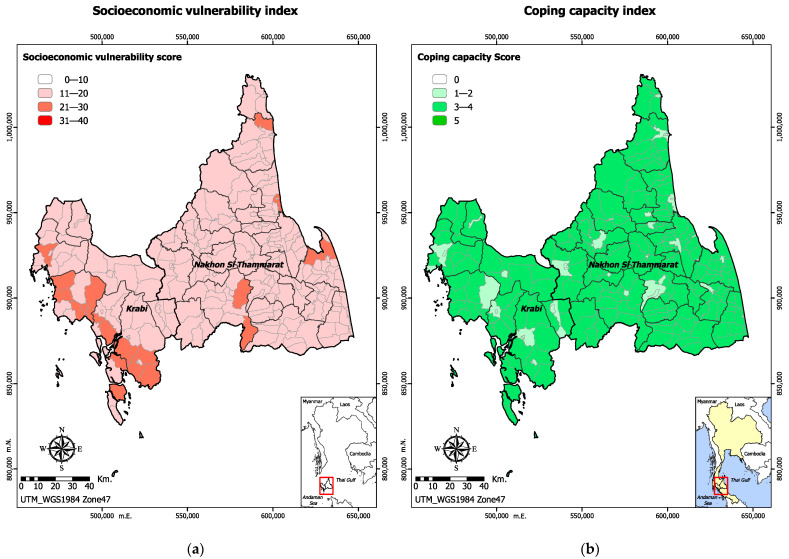
Resilience to natural hazards: (**a**) socio-economic vulnerability index; (**b**) coping capacity index.

Based on the variables used to assess vulnerability, a socio-economic vulnerability index was constructed and mapped. While vulnerability scores differed from one district to the other, more areas within Krabi were more vulnerable to the effects of hydro-meteorological hazards. Most districts in both provinces had socio-economic vulnerability scores ranging from 11 to 20, suggesting greater resilience as shown in Figure 5a.

Combining the soft and hard coping capacities, we derived coping capacity index scores. The average coping capacity index score across subdistricts in Krabi was 3 out of a possible 4, meaning that most of the subdistricts were largely resilient. In Nuea Khlong district, Khlong Khanan subdistrict had the highest coping capacity index score possible. In Nakhon Si Thammarat, the average coping capacity index score across subdistricts was also 3 out of a possible 4, with variations within subdistricts. In four of the districts, there were subdistricts with the highest coping capacity index scores of 4 out of 4. In both Krabi and Nakhon Si Thammarat provinces, most subdistricts had high coping capacity index scores as shown in Figure 5b. Few subdistricts recorded coping capacity index scores between 1 and 2 out of 4.

## 4. Discussion

The study conducted a quantitative socio-economic assessment of the resilience and coping capacities of communities in two coastal provinces, Krabi and Nakhon Si Thammarat, regarding hydrometeorological hazards. The study suggested that more areas within Krabi than Nakhon Si Thammarat province were more vulnerable to the effects of hydrometeorological hazards. This could be explained by the fact that most subdistricts in Krabi were more densely populated with higher proportions of children under 5 and adults compared to subdistricts in Nakhon Si Thammarat province.

Results showed variations in total socio-economic resilience index scores at subdistrict level. District averages of socio-economic resilience scores tend to mask the variations at subdistrict level. Similar findings were reported in an earlier study [6], and a recommendation was put forward to conduct more studies with rigorous methodologies at microlevel, i.e., at village or neighborhood level to get a nuanced understanding of community resilience.

In both Krabi and Nakhon Si Thammarat provinces, few subdistricts recorded coping capacity index scores between 1 and 2 out of 4. This was even though all subdistricts had a literacy rate between 90% and 95% among people aged 6 years and above. In addition, all subdistricts in both provinces were at least covered by either a functional hospital, either at community level, district level, or regional level. Future research could examine further the differences at more granular level to understand the variability in coping capacities by district.

The study had its own limitations. First, the study used overall literacy rates among people aged 6 years and above as the sole proxy indicator for communities’ soft coping capacities. Literacy rates at regional level were applied at subdistrict level, and this masks the actual variations. Literacy in terms of disaster response could be different, as it includes the application of local knowledge gained from experience based on past disaster exposure. Second, community resilience and coping capacities were assessed at subdistrict level. Future studies could focus on a more granular geographic scale to provide a more in-depth understanding on the variability of disaster resilience within the coastal provinces of Krabi and Nakhon Si Thammarat. Third, the study did not have a control area to use as a comparison that all study areas were prone to flooding.

## 5. Conclusions

In conclusion, the study makes explicit the realization that overall resilience at a macrolevel masks the greater variations at granular, localized geographical scales. Accurate measurement of a community’s circumstances before an adverse event is pre-requisite toward enhanced knowledge of a community’s capacity to adapt, respond to, and recover from hydrometeorological hazards. The challenge in update and apply lessons learnt either from historical experience or current insight are key points. Making better use across existing research is important for understanding the resilience to floods.

## Figures and Tables

**Table 1:** Number of administrative units by province.

Unit	Krabi (8 Districts)	Nakhon Si Thammarat (23 Districts)
Subdistrict	48	131
Subdistrict municipality	12	49
Town municipality	1	3
City municipality	0	1

**Table 2:** Variables used to measure disaster resilience.

Sub- Indicators	Variables	Definition ^1^	Data Sources (Year) ^2^
Exposure	Population density	Population density (scale 0 to 5) 0 = 0 1 = 1–200 people per square kilometer 2 = 201–400 people per square kilometer 3 = 401–600 people per square kilometer 4 601–800 people per square kilometer 5 > 800 people per square kilometer	DOPA (2017)
Vulnerability	Percentage of infant	Percentage of infant (scale 0 to 5) 0 = 0% 1 = 0.1–0.4% 2 = 0.5–0.8% 3 = 0.9–1.2% 4 = 1.3–1.7% 5 > 1.7%	DOPA (2017)
	Percentage of children under the age of five	Percentage of children under the age of five (scale 0 to 5) 0 = 0% 1 = 0.1–2.5% 2 = 2.6–5.0% 3 = 5.1–7.5% 4 = 7.6–10.0% 5 > 10.0%	DOPA (2017)
	Percentage of elderly population (60+)	Percentage of elderly population (60+) (scale 0 to 5) 0 = 0% 1 = 0.1–5.0% 2 = 5.1–10.0% 3 = 10.1–15.0% 4 = 15.1–20.0% 5 > 20.0%	DOPA (2017)
	Number of prisoners	Number of prisoners that exposed or at risk from disasters (scale 0 to 5) 0 = 0 1 = 1–1000 prisoners 2 = 1001–2000 prisoners 3 = 2001–3000 prisoners 4 = 3001–4000 prisoners 5 > 4000 prisoners	DOC (2018)
	Number of orphans and homeless persons	Number of orphans and homeless persons that exposed or at risk from disasters (scale 0 to 5) 0 = 0 1 = 1–100 persons 2 = 101–200 persons 3 = 201–300 persons 4 = 301–400 persons 5 > 400 persons	DCY, DSDW, DJOP (2019)
	Number of disabled persons	Number of disabled persons that exposed or at risk from disasters (scale 0 to 5) 0 = 0 1 = 1–250 persons 2 = 251–500 persons 3 = 501–750 persons 4 = 751–1000 persons 5 > 1000 persons	DSDW, Krabi Provincial PHO, Nakhon Si Thammarat Provincial PHO (2019)
	Prevalence of chronic diseases	Prevalence of chronic diseases include CKD, COPD, DM, HT, DM+HT, stroke (scale 0 to 5) 0 = 0 1 = total disease prevalence 0.1–3.5 2 = total disease prevalence 3.6–7.0 3 = total disease prevalence 7.1–10.5 4 = total disease prevalence 10.6–14.0 5 = total disease prevalence > 14.0	Krabi Provincial PHO, Nakhon Si Thammarat Provincial PHO (2019)
	Total area of mangrove forest	Total area of mangrove forest (scale 0 to 5) 0 = 0 rai 1 = 1–3000 rai 2 = 3001–6000 rai 3 = 6001–9000 rai 4 = 9001–12000 rai 5 > 12,000 rai	DMCR (2018)
Soft coping capacity	Literacy rate	Literacy rate (scale 0 to 1) 0 < 90% 1 ≥ 90%	NSO (2018)
Hard coping capacy	Hospital at subdistrict level	Hospital at subdistrict level (scale 0 to 1) 0 = No 1 = Yes	Strategy and Planning Division, Office of the Permanent Secretary of MoPH (2019)
	Hospital at district and provincial level	Hospital at district and provincial level (scale 0 to 1) 0 = No 1 = Yes	Strategy and Planning Division, Office of the Permanent Secretary of MoPH (2019)
	Telecommunication development	Information and communication technology master plan at SAO and municipality level (scale 0 to 1) 0 = No 1 = Yes	DLA, SAOs, Municipalities (2019)

^1^ CKD, chronic kidney disease; COPD, chronic obstructive pulmonary disease; DM, diabetes mellitus; HT, hypertension; SAO, Subdistrict Administration Organization. ^2^ DOPA, Department of Provincial Administration, Ministry of Interior; DOC, Department of Corrections, Ministry of Justice; DCY, Department of Children and Youth, Ministry of Social Development and Human Security; DSDW, Department of Social Development and Welfare, Ministry of Social Development and Human Security; DJOP, Department of Juvenile Observation and Protection, Ministry of Justice; PHO, Public Health Office, Ministry of Public Health; DMCR, Department of Marine and Coastal Resources, Ministry of Natural Resources and Environment; NSO, National Statistical Office; MoPH, Ministry of Public Health; DLA, Department of Local Administration, Ministry of Interior; SAOs, Subdistrict Administration Organizations.

## Data Availability

Not applicable.
